# Serial Magnetic Resonance Arthrography for a Midsubstance Capsular Tear in a Patient With Traumatic Anterior Shoulder Instability

**DOI:** 10.7759/cureus.59247

**Published:** 2024-04-29

**Authors:** Kei Matsunaga, Satoshi Miyake, Teruaki Izaki, Terufumi Shibata, Takuaki Yamamoto

**Affiliations:** 1 Department of Orthopedic Surgery, Fukuoka University Faculty of Medicine, Fukuoka, JPN; 2 Department of Orthopedic Surgery, Fukuoka University Chikushi Hospital, Fukuoka, JPN

**Keywords:** inferior glenohumeral ligament, recurrent shoulder dislocation, traumatic anterior shoulder instability, magnetic resonance arthrography, midsubstance capsular tear

## Abstract

The natural history of midsubstance capsular tears (MCTs) is unclear. We herein describe a case of MCT observed using serial magnetic resonance (MR) arthrography. A 46-year-old woman presented with excessive external rotation of the left glenohumeral joint, resulting in an initial anterior dislocation of the left shoulder. She subsequently developed recurrent shoulder joint dislocations. MR arthrography revealed an MCT without a Bankart lesion three months after the initial dislocation. She opted for nonoperative treatment, but the shoulder instability did not improve. The second MR arthrography, nine months after the initial dislocation, showed no natural healing of the MCT. The third MR arthrography, 12 months after the initial dislocation, also showed no natural healing. Her shoulder instability remained persistent. The patient then decided to have surgery. Arthroscopy revealed a large capsular defect extending from the glenoid to the humeral head in the anterior inferior glenohumeral ligamentous complex. The MCT was repaired with the placement of nonabsorbable sutures in a side-to-side fashion. At the final follow-up, three years postoperatively, the patient had no anterior shoulder instability. The Rowe score was 100 points. MR arthrography showed good repair integrity of the MCT at one year postoperatively. Serial MR arthrography was useful for both the patient and the shoulder surgeon in considering the treatment of the MCT, facilitating an accurate and qualitative assessment of whether natural healing of the MCT had been achieved.

## Introduction

The primary cause of traumatic anterior shoulder instability is functional, structural tears of the labrum-inferior glenohumeral ligament complex (LA-IGHLC), which is part of the articular capsule. Most of these lesions are avulsions of the labrum from the glenoid rim (Bankart lesions). Less frequently, midsubstance capsular tears (MCTs) also occur [1,2]. MCT is a rare cause of traumatic anterior shoulder instability [1,2]. Previous studies have shown that MCT often occurs at the time of the initial dislocation [3]. Because there are no reports of MCTs observed on serial magnetic resonance (MR) arthrography, the percentage of MCTs that achieve natural healing when the shoulder joint is dislocated for the first time remains unknown. We herein describe a case of MCT observed using serial MR arthrography. This article was previously presented as an abstract at the 146th West-Japanese Society of Orthopedics and Traumatology Meeting on November 12, 2022.

## Case presentation

A 46-year-old woman presented with excessive external rotation of the left glenohumeral joint, resulting in an initial anterior dislocation of the left shoulder. She subsequently developed recurrent shoulder joint dislocations. She was referred to our institution three months after the initial dislocation.

Physical examination revealed no limitation of joint range of motion, but severe anterior instability of the shoulder joint was present. The anterior apprehension test was positive, the sulcus test was negative, and the posterior apprehension test was negative. The Beighton score was 2 of 9 points. The Rowe score was 15 points, and the Western Ontario Shoulder Instability (WOSI) Index was 1,773 points. The score on the Japanese Society for Surgery of the Hand version of the Disability of the Arm, Shoulder, and Hand questionnaire (DASH score) was 62.5 points. The visual analog scale pain scores were 0 mm at rest, 87 mm at night, and 56 mm with activity (range: 0-100 mm). Plain radiographs and three-dimensional computed tomography scans showed no glenoid rim bone defect or Hill-Sachs lesions.

**Figure 1:**
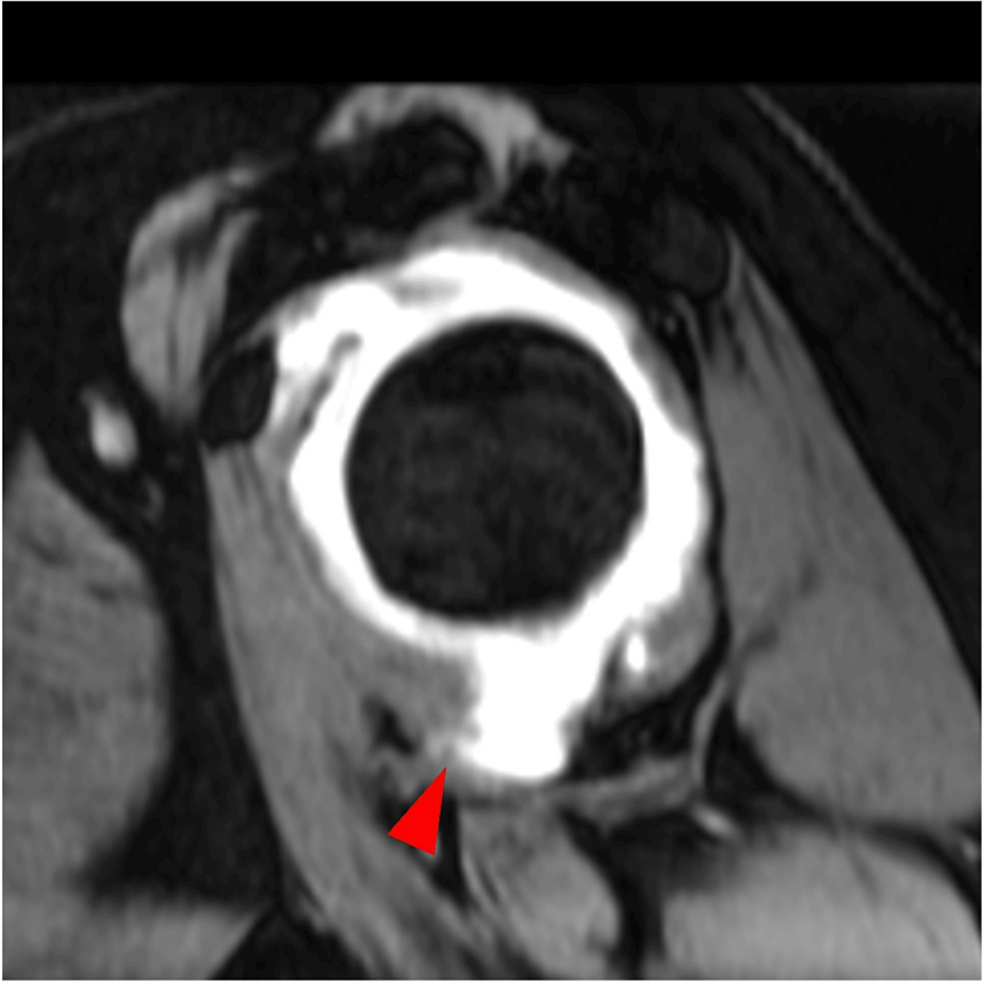
Oblique MR arthrography three months after initial shoulder dislocation Part of the joint capsule shows a distorted lateral convexity (i.e., an irregular outline sign) (arrowhead). MR, magnetic resonance

MR arthrography was performed, and the oblique sagittal view revealed a distorted shape of the lateral convexity in the MCT (Figure 1).

**Figure 2:**
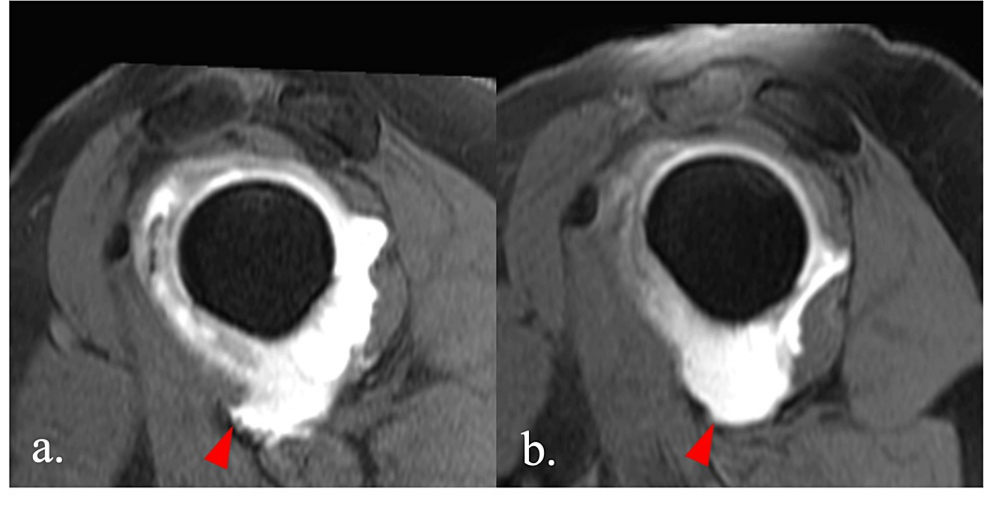
Oblique sagittal MR arthrography (a) nine months and (b) 12 months after the initial shoulder dislocation The arrowhead in (a) indicates that the irregular outline sign is still present. The arrowhead in (b) indicates that the irregular outline sign has increased in size. MR, magnetic resonance

There was no Bankart lesion or rotator cuff tear. Based on these physical and imaging findings, the patient was diagnosed with traumatic anterior shoulder instability due to an MCT. Surgical treatment was recommended because the patient’s shoulder instability was interfering with her daily activities. However, the patient opted for conservative treatment out of fear of surgery. Nine months after the initial shoulder dislocation, oblique sagittal MR arthrography showed that the distorted shape of the lateral convexity of the capsule had not improved, suggesting that the MCT had not healed naturally (Figure 2a). Twelve months after the initial shoulder dislocation, the distorted shape of the lateral convexity of the capsule had increased in size, as shown by oblique sagittal MR arthrography (Figure 2b).

**Figure 3:**
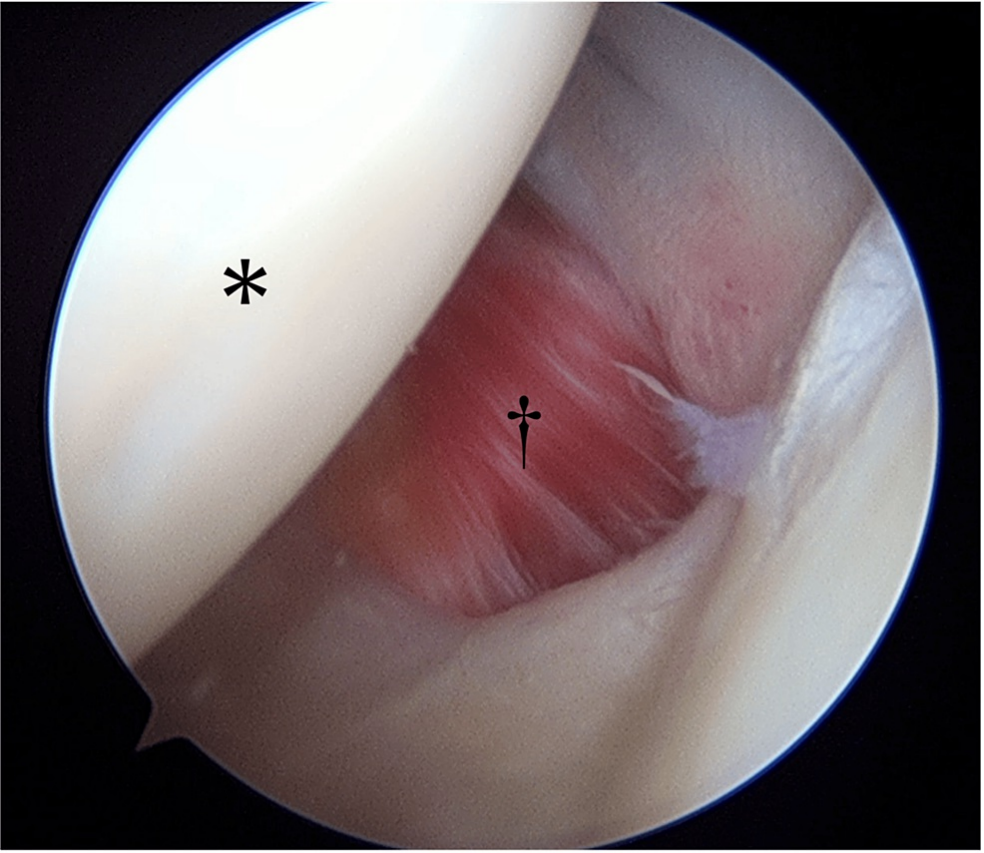
Arthroscopic view (posterior portal) ^*^ Humeral head; ^†^ MCT The subscapularis muscle is visible through the tear. MCT, midsubstance capsular tear

Meanwhile, the patient continued to experience instability in her left shoulder, which interfered with her daily activities. She decided to undergo arthroscopic shoulder surgery. Arthroscopic surgery was performed under general anesthesia in the beach chair position. Arthroscopy revealed a large capsular defect extending from the glenoid to the humeral head in the anterior inferior glenohumeral ligamentous complex (Figure 3).

The labrum was found to be intact. No other pathology associated with anterior shoulder instability was identified. The muscle belly of the subscapularis tendon was identified as a large capsular defect. The MCT was considered the primary cause of the traumatic anterior shoulder instability. Two anterior portals and one portal at the 5 o’clock position were created as working portals. The MCT was repaired with the placement of nonabsorbable sutures in a side-to-side fashion. The affected extremity was immobilized with a sling for four weeks postoperatively. Pendulum exercises were started one week postoperatively. Passive range-of-motion exercises were started at three weeks postoperatively, and active range-of-motion exercises were started at four weeks postoperatively.

**Figure 4:**
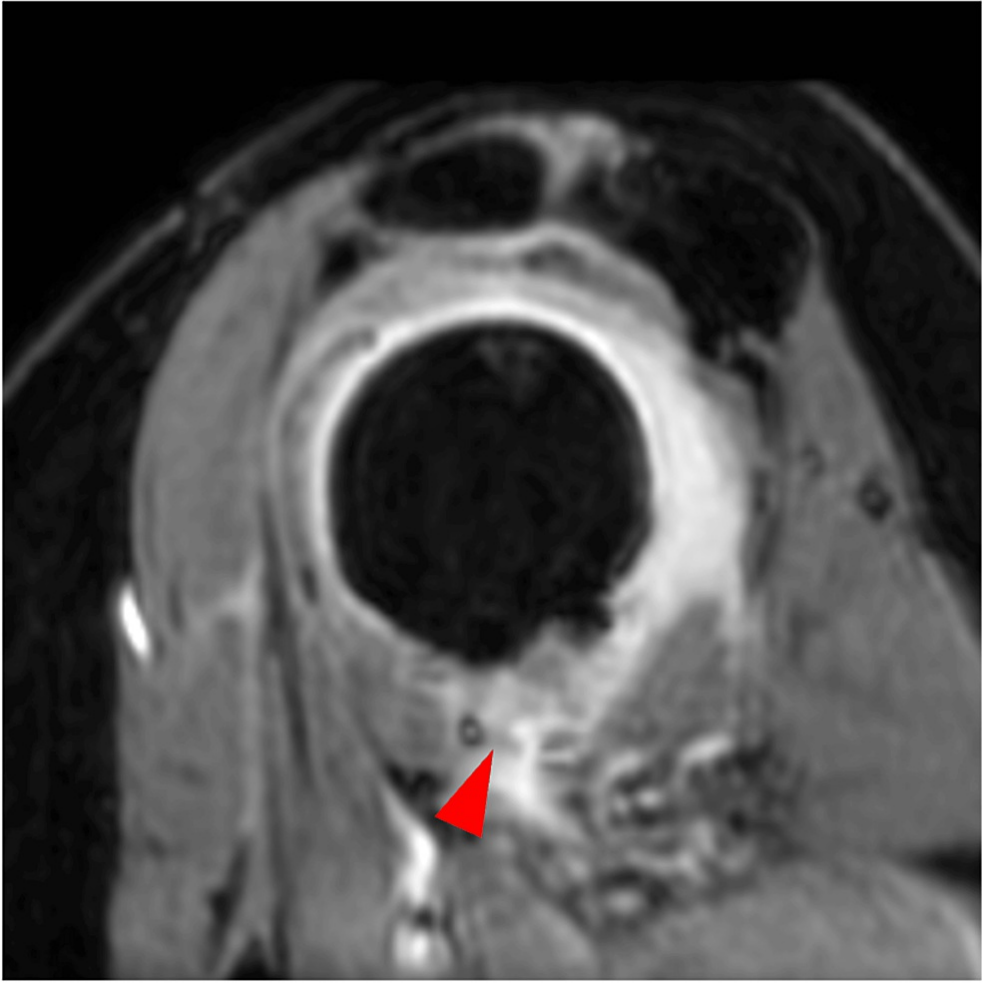
Oblique sagittal MR arthrography at one year postoperatively The arrowhead indicates the absence of the irregular outline sign. MR, magnetic resonance

At the final follow-up three years postoperatively, the patient had no anterior shoulder instability, the Rowe score was 100 points, the DASH score was 0 points, and the WOSI Index was 330 points. The visual analog scale pain scores were 0 mm at rest, at night, and with activity (range: 0-100 mm). MR arthrography imaging at one year postoperatively showed no distorted shape of the lateral convexity of the capsule (Figure 4).

## Discussion

Several investigators have reported the imaging features of MCT [4-6]. Rhee et al. retrospectively evaluated preoperative MR arthrography images in patients with arthroscopic findings of MCT [6]. Miyake et al. defined the distorted shape of the lateral convexity of the capsule margins on oblique sagittal MR arthrography as the “irregular outline sign” and reported this sign as an indicator of MCT [5]. In the present case, we used the same imaging features in the oblique sagittal section of preoperative MR arthrography to diagnose MCT. Several studies have shown that MCT often occurs at the time of the initial shoulder dislocation [3,7,8]. Bigliani et al. found that the LA-IGHLC failed at the midsubstance in 35% of all cases during tensile testing with 48 fresh-frozen cadaveric shoulders [7]. McMahon et al. found that the LA-IGHLC failed at the midsubstance in 16% of all cases during tensile testing with 12 fresh-frozen cadaveric shoulders [8]. Baker et al. reported that the frequency of MCT at the time of initial shoulder dislocation was high at 13.3% (six of 45 shoulders) [3]. Orvets et al. reported that the frequency of MCT within six months of initial shoulder dislocation was 25% (11 of 44 shoulders) [9].

However, MCT is only a minor cause of traumatic anterior shoulder instability. Ogawa and Yoshida and Mizuno et al. reported a 1.5% and 3.9% frequency, respectively [1,2]. Despite the high rate of MCT at the time of initial dislocation, the actual rate of traumatic anterior shoulder instability requiring surgery is low. It has been hypothesized that most MCTs that occur at the time of initial shoulder dislocation will heal naturally. However, this hypothesis remains unproven because no reports of MCTs have been followed by serial imaging.

Murphy et al. [10] performed a serial MR evaluation of humeral avulsions of the glenohumeral ligament (HAGL) lesions and reported that the lesions resolved in all three of their patients. The authors noted that HAGL lesions may heal naturally because of the vascular structure of the capsule and asserted the importance of serial imaging evaluation. They were unable to confirm the HAGL lesions arthroscopically. In the present case, we followed the MCT lesion with MR arthrography and finally confirmed the lesion arthroscopically. The MCT was observed as an irregular outline sign on the first MR arthrography, and this sign remained on the second MR arthrography. On the third MR arthrography, the irregular outline sign had increased in size (nine months after the first examination). This suggests that the MCT did not heal naturally within a certain period of time and may have remained. The visual information provided by serial MR arthrography indicating that the MCT did not heal naturally helped the patient make the decision to undergo surgery despite the fact that she had initially refused surgical treatment. It also assisted the shoulder surgeon in formulating a treatment plan. At the three-year postoperative follow-up, the patient was satisfied with her decision to undergo surgery because she had experienced no recurrence of shoulder instability and good function had been maintained. This is the first report describing the serial imaging evaluation of MCT using oblique sagittal MR arthrography. This case might be valuable to shoulder surgeons treating traumatic anterior shoulder instability by providing a partial picture of the natural history of MCT.

## Conclusions

This is the first report of a serial imaging evaluation of MCT using oblique sagittal MR arthrography. The MCT lesion was monitored by MR arthrography and ultimately confirmed by arthroscopy. Serial imaging assessment of MCT using MR arthrography provides advantages for both the patient and the shoulder surgeon in developing a treatment plan for MCT.
